# *cis*-Dichlorido[2-(3,5-dimethyl-1*H*-pyrazol-1-yl-κ*N* ^2^)ethanamine-κ*N*]palladium(II) dichloro­methane monosolvate

**DOI:** 10.1107/S1600536812032308

**Published:** 2012-07-21

**Authors:** Collins Obuah, James Darkwa, Alfred Muller

**Affiliations:** a Department of Chemistry, University of Johannesburg (APK Campus), PO Box 524, Auckland Park, Johannesburg, 2006, South Africa

## Abstract

In the title compound, [PdCl_2_(C_7_H_13_N_3_)]·CH_2_Cl_2_, the 2-(3,5-dimethyl-1*H*-pyrazol-1-yl)ethanamine ligand chelates the Pd^II^ atom *via* two N atoms forming a six-membered ring resulting in a distorted square-planar metal coordination environment, highlighted by N—Pd—Cl angles of 172.63 (8) and 174.98 (9)°. In addition to N—H⋯Cl hydrogen bonds creating infinite chains along [001], several C—H⋯Cl inter­actions are observed in the crystal structure.

## Related literature

For the synthesis and catalytic applications of Schiff base complexes, see: Connor *et al.* (2003 ▶); Wang *et al.* (1998 ▶). For catalytic hydrolysis of free and bound imines, see: Nolan & Hay (1974 ▶); Satchell & Satchell (1979 ▶); Hay (1987 ▶); Bähr & Thämlitz (1955 ▶); Bähr & Döge (1957 ▶). 
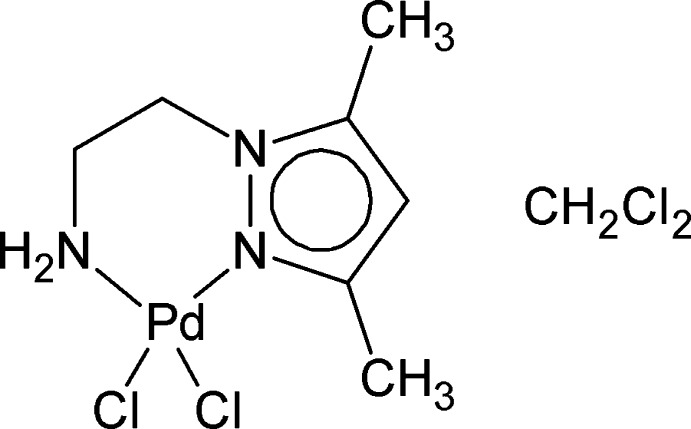


## Experimental

### 

#### Crystal data

[PdCl_2_(C_7_H_13_N_3_)]·CH_2_Cl_2_
*M* *_r_* = 401.43
Monoclinic, 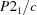

*a* = 11.9415 (11) Å
*b* = 11.432 (1) Å
*c* = 10.5901 (10) Å
β = 97.087 (2)°
*V* = 1434.7 (2) Å^3^
*Z* = 4
Mo *K*α radiation
μ = 2.02 mm^−1^
*T* = 100 K
0.31 × 0.27 × 0.26 mm

#### Data collection

Bruker X8 APEXII 4K KappaCCD diffractometer
Absorption correction: multi-scan (*SADABS*; Bruker, 2008 ▶) *T*_min_ = 0.574, *T*_max_ = 0.622
18522 measured reflections
3582 independent reflections
3373 reflections with *I* > 2σ(*I*)
*R*_int_ = 0.021

#### Refinement

*R*[*F* ^2^ > 2σ(*F* ^2^)] = 0.032
*wR*(*F* ^2^) = 0.085
*S* = 1.11
3582 reflections
155 parameters
2 restraints
H atoms treated by a mixture of independent and constrained refinement
Δρ_max_ = 2.28 e Å^−3^
Δρ_min_ = −1.12 e Å^−3^

### 

Data collection: *APEX2* (Bruker, 2011 ▶); cell refinement: *SAINT* (Bruker, 2008 ▶); data reduction: *SAINT* and *XPREP* (Bruker, 2008 ▶); program(s) used to solve structure: *SIR97* (Altomare *et al.*, 1999 ▶); program(s) used to refine structure: *SHELXL97* (Sheldrick, 2008 ▶); molecular graphics: *DIAMOND* (Brandenburg & Putz, 2005 ▶); software used to prepare material for publication: *WinGX* (Farrugia, 1999 ▶).

## Supplementary Material

Crystal structure: contains datablock(s) global, I. DOI: 10.1107/S1600536812032308/bt5968sup1.cif

Structure factors: contains datablock(s) I. DOI: 10.1107/S1600536812032308/bt5968Isup2.hkl

Additional supplementary materials:  crystallographic information; 3D view; checkCIF report

## Figures and Tables

**Table 1:** Hydrogen-bond geometry (Å, °)

*D*—H⋯*A*	*D*—H	H⋯*A*	*D*⋯*A*	*D*—H⋯*A*
N3—H3*B*⋯Cl1^i^	0.87 (2)	2.51 (4)	3.230 (3)	141 (5)
N3—H3*B*⋯Cl2^i^	0.87 (2)	2.66 (5)	3.297 (3)	131 (5)
N3—H3*A*⋯Cl3	0.86 (2)	2.79 (2)	3.631 (3)	169 (4)
C4—H4*A*⋯Cl2^ii^	0.99	2.76	3.720 (3)	163
C4—H4*A*⋯Cl2^ii^	0.99	2.76	3.720 (3)	163
C7—H7*A*⋯Cl1	0.98	2.74	3.455 (4)	130
C8—H8*A*⋯Cl2	0.99	2.71	3.428 (4)	130

Symmetry codes: (i) 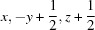; (ii) 
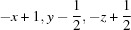
.

**Fig. 1.**
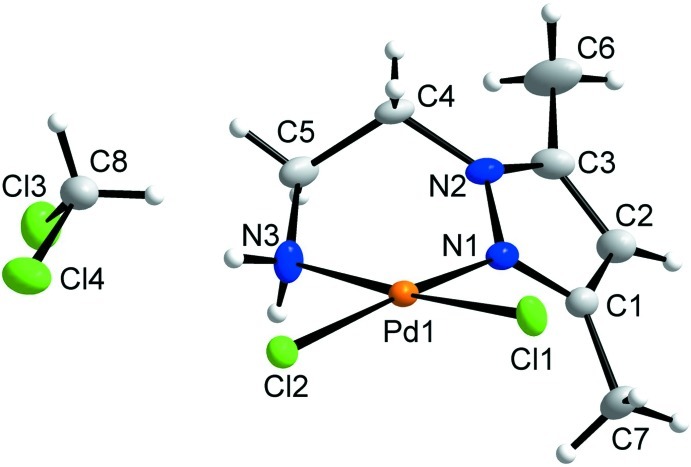
View of (I). Displacement ellipsoids are drawn at a 50% probability level.

**Fig. 2.**
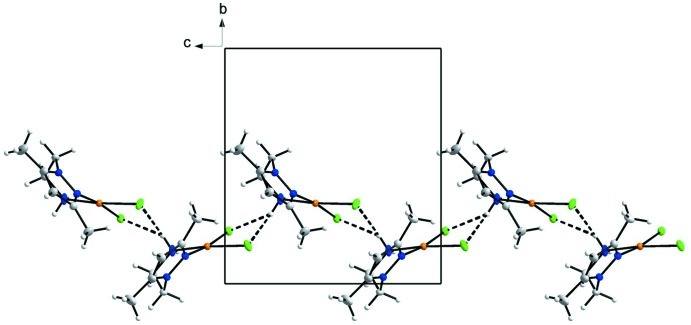
Packing diagram of (I) showing the infinite one-dimensional chains created along the [001] direction.

## supplementary crystallographic information

### Comment

Schiff base complexes have been synthesized and used in a number of catalytic reactions (Connor *et al.*, 2003; Wang *et al.*, 1998). Their popularity stems from their ease of synthesis, their ability to stabilize metals in different oxidation states and the ability to modify both the electronic and steric properties of the ligand. However, several reports of metal ions catalyzing the hydrolysis of free imines (Nolan & Hay, 1974; Satchell & Satchell, 1979; Hay, 1987) and bound imines (Bähr & Thämlitz, 1955; Bähr & Döge, 1957) have been reported. In an attempt to prepare [{2-(3,5-di-methylpyrazol-1-yl)-ethyl-(ferrocenylmethyl-methylene)-imine}PdCl_2_] as olefin transformation catalyst, we found the title compound 1 formed as a result of hydrolysis of the imine by the Pd(II) starting material.

The title compound 1 (Figure 1) *cis*-[(3,5-di-methylpyrazol-1-yl-ethylamine)PdCl_2_] crystallizes in the *P*2_1_/*c* (*Z*=4) space group with an accompanying CH_2_Cl_2_ solvate. Chelation of the pyrazolylamine to the metal coordination environment is distorted, highlighted by the dihedral angle of 7.75 (8)° between the Cl1—Pd–Cl2 and N1—Pd—N3 planes. The N—Pd—Cl angles of 172.63 (8) and 174.98 (9)° are further evidence of this distortion. Pd—Cl bond distances of 2.3070 (7) and 2.3004 (7) differ marginally, possibly due to the different electronic capabilities of the donor atoms of the pyrazolylamine.

Several N/C···Cl interactions are observed (see table 1), creating infinite one-dimensional chains along the [001] direction as shown in Figure 2.

### Experimental

A CH_2_Cl_2_ solution (20 ml) of 2-(3,5-di-methylpyrazol-1-yl)-ethyl-(ferrocenylmethyl-methylene)-imine (0.10 g, 0.28 mmol) was added to [PdCl_2_(CNMe)_2_] (0.07 g, 0.28 mmol) in CH_2_Cl_2_ (10 ml) while stirring. The resulting mixture was stirred for 18 h at 25 °C after, which hexane was added to precipitate an orange solid. The solid was filtered, washed three times with hexane and dried in air.

Light yellow single crystals were grown by slow evaporation at room temperature in CH_2_Cl_2_. Yield = 0.07 g, 80%.

^1^H NMR (CDCl_3_): δ 2.37 (s, 3H, CH_3_); 2.58 (s, 3H, CH_3_); 2.36 (s, 3H, CH_3_); 4.37 (s, 4H, CH_2_); 5.84 (s, 1H, pz). IR (Diamond ATR, cm^-1^): 3435 ν(NH)

### Refinement

All hydrogen atoms for methylene, methyl and aromatic H atoms were positioned in geometrically idealized positions with C—H = 0.99 Å, 0.98 Å and 0.95 Å respectively. All these hydrogen atoms were allowed to ride on their parent atoms with *U*_iso_(H) = 1.2*U*_eq_, except for methyl where *U*_iso_(H) = 1.5*U*_eq_ was utilized. The initial positions of methyl hydrogen atoms were located from a Fourier difference map and refined as fixed rotor. The amine hydrogen atoms were obtained from a Fourier difference map and restrained with the standard N—H distance of 0.87 Å. The highest residual peak and hole are 2.28 and -1.12 Å^-3^ respectively, both within 1 Å from Pd1 and represent no physical meaning.

### Crystal data

**Table:**

[PdCl_2_(C_7_H_13_N_3_)]·CH_2_Cl_2_	*F*(000) = 792
*M**_r_* = 401.43	*D*_x_ = 1.859 Mg m^−^^3^
Monoclinic, *P*2_1_/*c*	Mo *K*α radiation, λ = 0.71073 Å
Hall symbol: -P 2ybc	Cell parameters from 9923 reflections
*a* = 11.9415 (11) Å	θ = 2.5–28.4°
*b* = 11.432 (1) Å	µ = 2.02 mm^−^^1^
*c* = 10.5901 (10) Å	*T* = 100 K
β = 97.087 (2)°	Cube, orange
*V* = 1434.7 (2) Å^3^	0.31 × 0.27 × 0.26 mm
*Z* = 4	

### Data collection

**Table:**

Bruker X8 APEXII 4K KappaCCD diffractometer	3582 independent reflections
Graphite monochromator	3373 reflections with *I* > 2σ(*I*)
Detector resolution: 8.4 pixels mm^-1^	*R*_int_ = 0.021
ω & φ scans	θ_max_ = 28.4°, θ_min_ = 1.7°
Absorption correction: multi-scan (*SADABS*; Bruker, 2008)	*h* = −15→15
*T*_min_ = 0.574, *T*_max_ = 0.622	*k* = −15→14
18522 measured reflections	*l* = −14→14

### Refinement

**Table:**

Refinement on *F*^2^	Primary atom site location: structure-invariant direct methods
Least-squares matrix: full	Secondary atom site location: difference Fourier map
*R*[*F*^2^ > 2σ(*F*^2^)] = 0.032	Hydrogen site location: inferred from neighbouring sites
*wR*(*F*^2^) = 0.085	H atoms treated by a mixture of independent and constrained refinement
*S* = 1.11	*w* = 1/[σ^2^(*F*_o_^2^) + (0.0378*P*)^2^ + 4.6095*P*] where *P* = (*F*_o_^2^ + 2*F*_c_^2^)/3
3582 reflections	(Δ/σ)_max_ < 0.001
155 parameters	Δρ_max_ = 2.28 e Å^−^^3^
2 restraints	Δρ_min_ = −1.12 e Å^−^^3^

### Special details

**Table:**

Experimental. The intensity data was collected on a Bruker X8 Apex II 4 K Kappa CCD diffractometer using an exposure time of 10 s/frame. A total of 1491 frames were collected with a frame width of 0.5° covering up to θ = 28.40° with 99.5% completeness accomplished.
Geometry. All e.s.d.'s (except the e.s.d. in the dihedral angle between two l.s. planes) are estimated using the full covariance matrix. The cell e.s.d.'s are taken into account individually in the estimation of e.s.d.'s in distances, angles and torsion angles; correlations between e.s.d.'s in cell parameters are only used when they are defined by crystal symmetry. An approximate (isotropic) treatment of cell e.s.d.'s is used for estimating e.s.d.'s involving l.s. planes.
Refinement. Refinement of *F*^2^ against ALL reflections. The weighted *R*-factor *wR* and goodness of fit *S* are based on *F*^2^, conventional *R*-factors *R* are based on *F*, with *F* set to zero for negative *F*^2^. The threshold expression of *F*^2^ > σ(*F*^2^) is used only for calculating *R*-factors(gt) *etc*. and is not relevant to the choice of reflections for refinement. *R*-factors based on *F*^2^ are statistically about twice as large as those based on *F*, and *R*- factors based on ALL data will be even larger.

### Fractional atomic coordinates and isotropic or equivalent isotropic displacement parameters (Å^2^)

**Table:**

	*x*	*y*	*z*	*U*_iso_*/*U*_eq_	
Pd1	0.560565 (18)	0.158108 (19)	0.080787 (19)	0.01469 (8)	
Cl1	0.64115 (6)	0.16169 (7)	−0.10627 (7)	0.02358 (16)	
Cl2	0.38953 (6)	0.22298 (6)	−0.01819 (6)	0.01848 (14)	
Cl3	0.18032 (8)	0.10624 (10)	0.19406 (9)	0.0377 (2)	
Cl4	0.07255 (8)	0.14275 (8)	−0.06665 (10)	0.0362 (2)	
N1	0.7084 (2)	0.1182 (2)	0.1833 (2)	0.0174 (5)	
N2	0.7127 (2)	0.0281 (2)	0.2683 (2)	0.0187 (5)	
N3	0.4849 (2)	0.1402 (3)	0.2426 (2)	0.0211 (5)	
C1	0.8094 (3)	0.1697 (3)	0.1996 (3)	0.0190 (6)	
C2	0.8790 (3)	0.1109 (3)	0.2939 (3)	0.0241 (6)	
H2	0.9555	0.1286	0.3233	0.029*	
C3	0.8152 (3)	0.0221 (3)	0.3364 (3)	0.0243 (6)	
C4	0.6096 (3)	−0.0357 (3)	0.2823 (3)	0.0209 (6)	
H4A	0.6271	−0.1015	0.3424	0.025*	
H4B	0.5782	−0.0688	0.199	0.025*	
C5	0.5224 (3)	0.0435 (3)	0.3313 (3)	0.0242 (6)	
H5A	0.456	−0.004	0.3463	0.029*	
H5B	0.5548	0.0771	0.414	0.029*	
C6	0.8449 (4)	−0.0700 (4)	0.4360 (4)	0.0400 (9)	
H6A	0.803	−0.0556	0.5084	0.06*	
H6B	0.9261	−0.0671	0.4647	0.06*	
H6C	0.8251	−0.1473	0.4	0.06*	
C7	0.8348 (3)	0.2783 (3)	0.1295 (3)	0.0270 (7)	
H7A	0.822	0.2636	0.0377	0.041*	
H7B	0.9137	0.3008	0.1539	0.041*	
H7C	0.7852	0.3417	0.1511	0.041*	
C8	0.1640 (3)	0.0524 (3)	0.0354 (4)	0.0302 (7)	
H8A	0.2387	0.0491	0.0041	0.036*	
H8B	0.133	−0.0279	0.0341	0.036*	
H3A	0.4137 (17)	0.137 (4)	0.220 (4)	0.033 (12)*	
H3B	0.492 (5)	0.204 (3)	0.287 (5)	0.067 (19)*	

### Atomic displacement parameters (Å^2^)

**Table:**

	*U*^11^	*U*^22^	*U*^33^	*U*^12^	*U*^13^	*U*^23^
Pd1	0.01967 (12)	0.01323 (12)	0.01177 (11)	−0.00021 (7)	0.00431 (8)	0.00061 (7)
Cl1	0.0239 (3)	0.0321 (4)	0.0162 (3)	0.0084 (3)	0.0083 (3)	0.0089 (3)
Cl2	0.0209 (3)	0.0198 (3)	0.0152 (3)	0.0011 (2)	0.0042 (2)	−0.0005 (2)
Cl3	0.0388 (5)	0.0452 (6)	0.0314 (4)	0.0025 (4)	0.0136 (4)	0.0040 (4)
Cl4	0.0366 (5)	0.0254 (4)	0.0442 (5)	0.0005 (3)	−0.0050 (4)	−0.0017 (4)
N1	0.0235 (12)	0.0153 (12)	0.0141 (11)	−0.0003 (9)	0.0049 (9)	0.0015 (9)
N2	0.0282 (13)	0.0138 (12)	0.0140 (11)	−0.0024 (9)	0.0015 (9)	0.0015 (9)
N3	0.0184 (12)	0.0343 (16)	0.0110 (11)	0.0007 (10)	0.0033 (9)	0.0009 (10)
C1	0.0225 (14)	0.0177 (14)	0.0172 (13)	−0.0002 (11)	0.0042 (10)	0.0007 (11)
C2	0.0259 (15)	0.0223 (16)	0.0229 (14)	−0.0006 (12)	−0.0020 (11)	0.0023 (12)
C3	0.0333 (16)	0.0184 (15)	0.0195 (14)	0.0001 (12)	−0.0031 (12)	0.0015 (11)
C4	0.0334 (16)	0.0126 (13)	0.0174 (13)	−0.0052 (11)	0.0060 (11)	−0.0001 (10)
C5	0.0375 (17)	0.0193 (15)	0.0179 (13)	−0.0045 (13)	0.0110 (12)	0.0002 (11)
C6	0.049 (2)	0.031 (2)	0.036 (2)	−0.0079 (17)	−0.0139 (17)	0.0140 (16)
C7	0.0290 (16)	0.0234 (17)	0.0293 (16)	−0.0047 (13)	0.0062 (12)	0.0081 (13)
C8	0.0289 (16)	0.0224 (16)	0.0400 (19)	0.0001 (13)	0.0066 (14)	0.0009 (14)

### Geometric parameters (Å, º)

**Table:**

Pd1—N1	2.007 (3)	C2—H2	0.95
Pd1—N3	2.044 (3)	C3—C6	1.501 (5)
Pd1—Cl2	2.3004 (7)	C4—C5	1.519 (4)
Pd1—Cl1	2.3070 (7)	C4—H4A	0.99
Cl3—C8	1.777 (4)	C4—H4B	0.99
Cl4—C8	1.770 (4)	C5—H5A	0.99
N1—C1	1.335 (4)	C5—H5B	0.99
N1—N2	1.365 (3)	C6—H6A	0.98
N2—C3	1.344 (4)	C6—H6B	0.98
N2—C4	1.454 (4)	C6—H6C	0.98
N3—C5	1.484 (4)	C7—H7A	0.98
N3—H3A	0.855 (19)	C7—H7B	0.98
N3—H3B	0.87 (2)	C7—H7C	0.98
C1—C2	1.391 (4)	C8—H8A	0.99
C1—C7	1.496 (4)	C8—H8B	0.99
C2—C3	1.378 (5)		
			
N1—Pd1—N3	88.52 (10)	C5—C4—H4A	109.4
N1—Pd1—Cl2	172.63 (8)	N2—C4—H4B	109.4
N3—Pd1—Cl2	87.40 (8)	C5—C4—H4B	109.4
N1—Pd1—Cl1	92.08 (7)	H4A—C4—H4B	108
N3—Pd1—Cl1	174.98 (9)	N3—C5—C4	113.2 (2)
Cl2—Pd1—Cl1	92.52 (3)	N3—C5—H5A	108.9
C1—N1—N2	106.7 (2)	C4—C5—H5A	108.9
C1—N1—Pd1	133.7 (2)	N3—C5—H5B	108.9
N2—N1—Pd1	119.16 (19)	C4—C5—H5B	108.9
C3—N2—N1	110.5 (3)	H5A—C5—H5B	107.7
C3—N2—C4	130.2 (3)	C3—C6—H6A	109.5
N1—N2—C4	118.9 (2)	C3—C6—H6B	109.5
C5—N3—Pd1	118.4 (2)	H6A—C6—H6B	109.5
C5—N3—H3A	111 (3)	C3—C6—H6C	109.5
Pd1—N3—H3A	107 (3)	H6A—C6—H6C	109.5
C5—N3—H3B	106 (4)	H6B—C6—H6C	109.5
Pd1—N3—H3B	110 (4)	C1—C7—H7A	109.5
H3A—N3—H3B	103 (5)	C1—C7—H7B	109.5
N1—C1—C2	109.3 (3)	H7A—C7—H7B	109.5
N1—C1—C7	122.5 (3)	C1—C7—H7C	109.5
C2—C1—C7	128.1 (3)	H7A—C7—H7C	109.5
C3—C2—C1	106.6 (3)	H7B—C7—H7C	109.5
C3—C2—H2	126.7	Cl4—C8—Cl3	111.3 (2)
C1—C2—H2	126.7	Cl4—C8—H8A	109.4
N2—C3—C2	106.9 (3)	Cl3—C8—H8A	109.4
N2—C3—C6	122.3 (3)	Cl4—C8—H8B	109.4
C2—C3—C6	130.8 (3)	Cl3—C8—H8B	109.4
N2—C4—C5	111.1 (3)	H8A—C8—H8B	108
N2—C4—H4A	109.4		
			
N3—Pd1—N1—C1	−127.5 (3)	Pd1—N1—C1—C7	−3.4 (5)
Cl1—Pd1—N1—C1	57.5 (3)	N1—C1—C2—C3	−1.0 (4)
N3—Pd1—N1—N2	43.7 (2)	C7—C1—C2—C3	175.0 (3)
Cl1—Pd1—N1—N2	−131.3 (2)	N1—N2—C3—C2	−0.1 (4)
C1—N1—N2—C3	−0.5 (3)	C4—N2—C3—C2	−172.6 (3)
Pd1—N1—N2—C3	−173.9 (2)	N1—N2—C3—C6	−179.1 (3)
C1—N1—N2—C4	173.0 (3)	C4—N2—C3—C6	8.4 (5)
Pd1—N1—N2—C4	−0.4 (3)	C1—C2—C3—N2	0.7 (4)
N1—Pd1—N3—C5	−39.8 (2)	C1—C2—C3—C6	179.5 (4)
Cl2—Pd1—N3—C5	146.4 (2)	C3—N2—C4—C5	109.0 (4)
N2—N1—C1—C2	1.0 (3)	N1—N2—C4—C5	−63.0 (3)
Pd1—N1—C1—C2	172.9 (2)	Pd1—N3—C5—C4	−3.9 (4)
N2—N1—C1—C7	−175.4 (3)	N2—C4—C5—N3	63.1 (3)

### Hydrogen-bond geometry (Å, º)

**Table:**

*D*—H···*A*	*D*—H	H···*A*	*D*···*A*	*D*—H···*A*
N3—H3*B*···Cl1^i^	0.87 (2)	2.51 (4)	3.230 (3)	141 (5)
N3—H3*B*···Cl2^i^	0.87 (2)	2.66 (5)	3.297 (3)	131 (5)
N3—H3*A*···Cl3	0.86 (2)	2.79 (2)	3.631 (3)	169 (4)
C4—H4*A*···Cl2^ii^	0.99	2.76	3.720 (3)	163
C4—H4*A*···Cl2^ii^	0.99	2.76	3.720 (3)	163
C7—H7*A*···Cl1	0.98	2.74	3.455 (4)	130
C8—H8*A*···Cl2	0.99	2.71	3.428 (4)	130

Symmetry codes: (i) *x*, −*y*+1/2, *z*+1/2; (ii) −*x*+1, *y*−1/2, −*z*+1/2.

## References

[bb1] Altomare, A., Burla, M. C., Camalli, M., Cascarano, G. L., Giacovazzo, C., Guagliardi, A., Moliterni, A. G. G., Polidori, G. & Spagna, R. (1999). *J. Appl. Cryst.* **32**, 115–119.

[bb2] Bähr, G. & Döge, H. G. (1957). *Z. Anorg. Allg. Chem.* **292**, 119–138.

[bb3] Bähr, G. & Thämlitz, H. (1955). *Z. Anorg. Allg. Chem.* **282**, 3–11.

[bb4] Brandenburg, K. & Putz, H. (2005). *DIAMOND* Crystal Impact GbR, Bonn, Germany.

[bb5] Bruker (2008). *SADABS*, *SAINT* and *XPREP* Bruker AXS Inc., Madison, Wisconsin, USA.

[bb6] Bruker (2011). *APEX2* Bruker AXS Inc., Madison, Wisconsin, USA.

[bb7] Connor, E. F., Younkin, T. R., Henderson, J. I., Waltman, A. W. & Grubbs, R. H. (2003). *Chem. Commun.* pp. 2272–2273. 10.1039/b306701g 14518870

[bb8] Farrugia, L. J. (1999). *J. Appl. Cryst.* **32**, 837–838.

[bb9] Hay, R. W. (1987). *Comprehensive Coordination Chemistry.*, Vol. 6, p. 441. Oxford: Pergamon.

[bb10] Nolan, K. B. & Hay, R. W. (1974). *J. Chem. Soc. Dalton Trans.* pp. 914–920.

[bb11] Satchell, D. P. N. & Satchell, R. S. (1979). *Annu. Rep. Chem. Sect. A Inorg. Chem.* **75**, 25–48.

[bb12] Sheldrick, G. M. (2008). *Acta Cryst.* A**64**, 112–122. 10.1107/S0108767307043930 18156677

[bb13] Wang, C., Friedrich, S., Younkin, T. R., Li, R. T., Grubbs, R. H., Bansleben, D. A. & Day, M. W. (1998). *Organometallics*, **17**, 3149–3151.

